# Association of baseline and longitudinal plasma retinol-binding protein 4 with all-cause mortality in maintenance hemodialysis patients

**DOI:** 10.3389/fendo.2025.1434757

**Published:** 2025-08-13

**Authors:** Thung-Lip Lee, Chao-Ping Wang, Chin-Feng Hsuan, Chia-Chang Hsu, Yung-Chuan Lu, Wei-Hua Tang, Ching-Ting Wei, Ya-Ai Cheng, Fu-Mei Chung, Yau-Jiunn Lee, I-Ting Tsai

**Affiliations:** ^1^ Division of Cardiology, Department of Internal Medicine, E-Da Hospital, I-Shou University, Kaohsiung, Taiwan; ^2^ School of Medicine for International Students, College of Medicine, I-Shou University, Kaohsiung, Taiwan; ^3^ School of Medicine, College of Medicine, I-Shou University, Kaohsiung, Taiwan; ^4^ Division of Cardiology, Department of Internal Medicine, E-Da Dachang Hospital, I-Shou University, Kaohsiung, Taiwan; ^5^ Division of Gastroenterology and Hepatology, Department of Internal Medicine, E-Da Hospital, I-Shou University, Kaohsiung, Taiwan; ^6^ Health Examination Center, E-Da Dachang Hospital, I-Shou University, Kaohsiung, Taiwan; ^7^ The School of Chinese Medicine for Post Baccalaureate, College of Medicine, I-Shou University, Kaohsiung, Taiwan; ^8^ Division of Endocrinology and Metabolism, Department of Internal Medicine, E-Da Hospital, I-Shou University, Kaohsiung, Taiwan; ^9^ Division of Cardiology, Department of Internal Medicine, Taipei Veterans General Hospital, Yuli Branch, Hualien, Taiwan; ^10^ Faculty of Medicine, School of Medicine, National Yang Ming Chiao Tung University, Taipei, Taiwan; ^11^ Division of General Surgery, Department of Surgery, E-Da Hospital, I-Shou University, Kaohsiung, Taiwan; ^12^ Department of Health Care Administration, College of Medicine, I-Shou University, Kaohsiung, Taiwan; ^13^ Lee’s Endocrinologic Clinic, Pingtung, Taiwan; ^14^ Department of Emergency, E-Da Hospital, I-Shou University, Kaohsiung, Taiwan

**Keywords:** hemodialysis, retinol-binding protein 4, all-cause mortality, structural equation model, prognostic biomarker

## Abstract

**Introduction:**

Elevated levels of retinol-binding protein 4 (RBP4) have been linked to conditions including cardiovascular disease, type 2 diabetes, obesity, and insulin resistance. However, the associations between baseline and longitudinal RBP4 levels with all-cause mortality in maintenance hemodialysis patients remain uncertain. The aim of this study was to investigate this issue.

**Methods:**

A total of 342 consecutive patients undergoing maintenance hemodialysis at a single hemodialysis center between 2010 and 2022 were assessed. Enzyme-linked immunosorbent assay was used to quantify plasma RBP4 concentrations. Patient outcomes were assessed using Cox regression and Kaplan-Meier analyses.

**Results:**

Non-survivors were associated with significantly lower baseline and longitudinal RBP4 levels compared to the survivors. A trend toward reduced all-cause mortality was found across quartiles of baseline and longitudinal RBP4 levels (p for trend < 0.0001). Multivariate analysis showed that the patients in the lowest quartiles (Quartile 1) of baseline and longitudinal RBP4 levels had hazard ratios of 2.22 (95% CI: 1.16-4.31) and 2.44 (95% CI: 1.30-4.84) for all-cause mortality, respectively, compared to those in the fourth quartile. In contrast to the non-survivors, the survivors had significantly higher plasma RBP4 levels during the 1-year follow-up period compared to the levels at the initiation of hemodialysis (p = 0.001). In addition, a positive association was observed between baseline RBP4 concentration and albumin level, while negative associations were observed between baseline RBP4 concentration and levels of aspartate aminotransferase, high-sensitivity C-reactive protein, and mean corpuscular volume. Structural equation model analysis confirmed the effect of RBP4 on mortality.

**Discussion:**

Associations were identified between low baseline and longitudinal RBP4 plasma concentrations and all-cause mortality in our cohort of Taiwanese maintenance hemodialysis patients.

## Introduction

Chronic kidney disease (CKD) is an increasingly significant health concern worldwide, associated with elevated rates of morbidity and mortality, especially in those who progress to end-stage kidney disease (ESKD) (1, 2). Patients with ESKD are at increased susceptibility to atherosclerosis, cardiovascular disease (CVD), and infections, all of which increase the risk of mortality (3–5). A prior investigation demonstrated elevated levels of reactive oxygen species in ESKD patients with type 2 diabetes, and that this was associated with endothelial dysfunction. This phenomenon coupled with chronic inflammation can increase the progression of atherosclerosis, and consequently increase the risk of CVD (6). In addition to the known risk factors such as chronic hyperglycemia, higher uremic toxin and free fatty acid levels along with altered metabolism of vitamin A may also contribute to increased reactive oxygen species production, oxidative tissue damage and stress in patients with ESKD (7). Elevated plasma retinol concentrations have also been reported in kidney failure patients. Physiologically, the concentration of retinol is regulated by the hepatic synthesis of its specific carrier, retinol-binding protein 4 (RBP4), a small visceral protein (approximately 21 kDa). RBP4 delivers retinol to target tissues, and it is catabolized in the kidneys. Consequently, dysregulated catabolism of RBP4-retinol complexes in the kidneys due to renal failure can lead to the build-up and altered delivery of retinol to peripheral tissues in patients with ESKD (8).

RBP4 functions not only as a carrier of retinol but also as an adipokine. It is widely expressed in adipose tissue, and elevated concentrations have been causally linked to insulin resistance in animal models (9), and also closely associated with blood pressure levels (10), hypertriglyceridemia (11), and type 2 diabetes (12). Elevated RBP4 concentrations have also been associated with microalbuminuria (13), CKD (14), and clinical atherosclerosis (15) in individuals with type 2 diabetes. In contrast, Riccioni et al. reported that carotid atherosclerosis was significantly associated with a low plasma concentration of vitamin A in a small cohort of ESKD patients (16). In addition, Connolly et al. found that a low retinol concentration was associated with a higher risk of mortality in kidney transplant recipients (17). Espe et al. also found a robust association between low concentrations of retinol and RBP4 with both all-cause mortality and sudden cardiac death in hemodialysis patients with diabetes (18).

Taken together, research on the metabolism of RBP4 in hemodialysis patients has been inconclusive. Moreover, the relationship between RBP4 levels and survival among hemodialysis patients remains unclear. Therefore, in this study, we investigated potential associations between baseline and longitudinal RBP4 levels with all-cause mortality among maintenance hemodialysis patients. We also investigated the effects of RBP4 on all-cause mortality with a structural equation model (SEM).

## Materials and methods

### Participants and design of the study

We performed this prospective single-center study from August 1, 2010 to December 31, 2022 at Lee’s Endocrinology Clinic, Pingtung, Taiwan. Participants were included if they had undergone at least 3 months of hemodialysis and were ≥ 18 years of age. All dialysis patients included in this study were clinically stable, without acute symptoms or comorbidities unrelated to kidney disease. The exclusion criteria were: (I) history of kidney transplantation or peritoneal dialysis; (II) diagnosis of malignancy; (III) use of immunosuppressive drugs; (IV) loss to follow-up; and (V) development of acute infection within 3 months of initiating hemodialysis; and (VI) presence of other chronic progressive conditions such as heart failure or active systemic lupus erythematosus. Individuals unable or unwilling to provide informed consent were also excluded.

We also enrolled 185 individuals (99 males and 86 females; mean age, 59 ± 6 years) who underwent routine medical examinations at E-Da Hospital, none of whom had clinical evidence of any major diseases, as controls. All participants signed informed consent forms before inclusion into this study. The E-Da Hospital Human Research Ethics Committee granted approval for the study.

### Baseline definitions and characteristics

We obtained data on baseline demographics and risk factors for all-cause mortality from the hemodialysis database of Lee’s Endocrinology Clinic. The duration of hemodialysis, weight, height, sex, age and systolic/diastolic blood pressures before dialysis, along with comorbidities and etiology of ESKD were also recorded. A fasting plasma glucose concentration ≥ 126 mg/dL, prescriptions for anti-diabetic medications, or a diagnosis of diabetes were defined as indicating the presence of diabetes. Systolic/diastolic blood pressures ≥140/90 mmHg and prescriptions for anti-hypertensive medications were defined as indicating the presence of hypertension. A serum triglyceride level ≥ 150 mg/dL, low serum high-density lipoprotein cholesterol level (< 40 mg/dL in males and < 50 mg/dL in females), and prescriptions for anti-lipidemic medications were defined as indicating the presence of dyslipidemia.

### Laboratory measurements

Serum levels of phosphate, calcium, potassium, transferrin saturation, parathyroid hormone, triglycerides, total cholesterol, creatinine, albumin, glucose, ferritin, and uric acid were measured following standard protocols as reported previously (19). Hemoglobin (measured in g/dL), total leukocyte and red blood cell counts, hematocrit (measured in %), and mean corpuscular volume were evaluated with an automatic hematological analyzer (XE‐2100 Hematology Alpha Transportation System; Sysmex Corporation). Adequacy of dialysis was assessed using Kt/V (20). Serum aspartate aminotransferase (AST) and alanine aminotransferase (ALT) levels were also measured according to the Japanese Society of Clinical Chemistry protocols (21). We used the CKD-EPI formula to estimate the glomerular filtration rate, and Jaffe method for serum creatinine (22).

### Measurements of plasma RBP4 and high-sensitivity C-reactive protein

Baseline and longitudinal RBP4 concentrations were measured in blood samples taken at hemodialysis initiation and after 1 year of follow-up using an enzyme-linked immunosorbent assay kit (Quantikine Human RBP4 Immunoassay; R&D Systems, Minneapolis, MN). The intra- and inter-assay coefficients of variation for the assay were 5.7-8.1% and 5.8-8.6%, respectively (both n = 3). Plasma hs-CRP levels were measured with an immunochemical system (IMMAGE, Beckman Coulter, Immunochemistry Systems, La Brea, CA), with a detection limit of 0.2 mg/L. All measurements were performed in duplicate for each experiment.

### Outcomes

The primary outcome was all-cause mortality. The survival period was calculated from enrollment to death or censoring due to reasons such as transfer to another dialysis center, kidney transplant, loss to follow-up, or reaching the end of the study (December 31, 2022).

### Statistical analysis

Continuous variables are reported as mean (SD) or median (interquartile range), and categorical variables as frequency and percentage. Continuous variables were compared with the Student’s *t*-test, and the chi-square test was used for categorical variables. To account for the skewed distribution of triglycerides, parathyroid hormone, white blood cell count, hs-CRP, ferritin, and transferrin saturation, logarithmic transformation was applied prior to analysis. We used a generalized linear model and the Cochran-Armitage test to assess trends in both continuous and categorical variables across the quartiles of baseline RBP4 levels. Univariate and multivariate Cox proportional hazards analyses were performed to estimate hazard ratios (HRs) and 95% confidence intervals (CIs) for all-cause mortality in each quartile, with the highest quartile serving as the reference for both baseline and longitudinal RBP4. To test linear trends of risk level, quartile rank was treated as a continuous variable in the regression analysis. Additionally, interaction terms between baseline RBP4 and hs-CRP, and between baseline RBP4 and albumin, were incorporated into Cox regression models to assess potential effect modification. Subgroup analyses were also performed to examine the association between RBP4 levels and all-cause mortality stratified by sex (male vs. female), diabetes status (T2DM vs. non-T2DM), and the underlying cause of CKD (diabetic nephropathy, glomerulonephritis, and other etiologies). Hazard ratios for each quartile of RBP4 were calculated within each subgroup using univariate Cox regression models.

The Kaplan-Meier method and log-rank test were used to compare categories of baseline RBP4 stratified by quartile. Receiver operating characteristic (ROC) analysis was performed to determine the optimal cutoff point of baseline RBP4 for predicting all-cause mortality. Plasma levels of RBP4 were compared between baseline and longitudinal follow-up (after 1 year) using paired Student *t*-tests. Correlations between baseline RBP4 and other factors were evaluated with Pearson’s correlation analysis and simple linear regression analysis. Results were considered significant at p < 0.05. The statistical analyses were performed with JMP v7.0 for Windows (SAS Institute, Cary, NC).

In addition, we used IBM SPSS AMOS v24 (Amos Development Corp.) to construct the path model and SEM. Statistical fit of the models were assessed using standard criteria, including standardized root mean square residual (SRMSR) < 0.06, root mean square error of approximation (RMSEA) < 0.08, and a comparative fit index (CFI) > 0.90 (23). The maximum likelihood method was used to determine model fit. Standardized path coefficients were used to present the results along with their statistical significance. Finally, to assess the adequacy of our sample size, we performed a *post hoc* power analysis based on the observed hazard ratio of 2.24 for all-cause mortality in the lowest quartile of baseline RBP4 compared to the highest quartile. With 141 deaths among 342 patients, the estimated statistical power exceeded 99.99% at a two-sided alpha level of 0.05, indicating sufficient power to detect the observed association.

## Results

Of 420 hemodialysis patients screened, 78 were excluded for the following reasons: 5 due to a history of kidney transplantation, 7 with a history of peritoneal dialysis, 11 diagnosed with malignancy, 9 using immunosuppressive drugs, 31 lost to follow-up, and 15 who developed acute infections within 3 months of initiating hemodialysis. The remaining 342 patients were enrolled, and the median follow-up period was 4 years (interquartile range, 1 to 6 years).

### Baseline characteristics

The baseline plasma RBP4 levels were higher in the hemodialysis patients than in the 185 controls (mean ± SD: 89.0 ± 20.5 μg/mL vs. 29.2 ± 9.7 μg/mL, p < 0.0001, **Supplementary Figure 1**). The characteristics of the patients are shown in **Supplementary Table 1**. The mean ± SD age of the patients was 63.7 ± 13.5 years. The prevalence rates of hyperlipidemia, diabetes mellitus, and hypertension were 38.9%, 73.4%, and 64.3%, respectively, with a median duration of hemodialysis of 4 years. Regarding the etiology of ESKD, 195 (57.0%) patients had diabetic nephropathy, 42 (12.3%) had vascular hypertension, 74 (21.6%) had chronic glomerulonephritis, and 31 (9.1%) had other conditions.

### Baseline biochemical and clinical characteristics of the patients by survival status

The non-survivors were older, had a shorter duration of hemodialysis, and lower systolic blood pressure and body mass index compared to the survivors. In addition, the non-survivors had elevated estimated glomerular filtration rate (eGFR), AST, alkaline phosphatase, and hs-CRP, along with lower creatinine, phosphate, parathyroid hormone, albumin, baseline RBP4, and longitudinal RBP4 levels compared to the survivors (**Table 1**).

**Table 1:** Baseline clinical and biochemical characteristics of the study participants by survival status.

Variable	Death	Survival	p-value
No	141	201	
Age (years)	68.7 ± 12.2	60.2 ± 13.3	<0.0001
Male (n, %)	75(53.2)	113(56.2)	0.580
Time on hemodialysis (years)	3.0(1.0-5.0)	5.0(2.0-7.0)	<0.0001
Comorbidities (n, %)			
Hypertension	86(61.0)	139(69.2)	0.172
Diabetes mellitus	110(78.0)	142(70.7)	0.144
Hyperlipidemia	58(41.1)	78(38.8)	0.729
Etiology of ESRD (n, %)			
Diabetic nephropathy	85(60.3)	110(54.7)	0.307
Hypertensive nephropathy	17(12.1)	25(12.4)	0.916
Chronic glomerulonephritis	29(20.6)	45(22.4)	0.687
Others	10(7.1)	21(10.5)	0.287
Body mass index (kg/m^2^)	22.0 ± 3.7	23.8 ± 4.5	0.0001
Systolic blood pressure (mmHg)	142 ± 22	150 ± 22	0.001
Diastolic blood pressure (mmHg)	71 ± 12	73 ± 11	0.158
Total cholesterol (mg/dl)	167.2 ± 47.0	168.9 ± 39.8	0.728
Triglyceride (mg/dl)	107.0(80.0-166.0)	121.5(75.0-188.5)	0.074
Fasting glucose (mg/dl)	153.3 ± 86.2	138.5 ± 73.3	0.096
Uric acid (mg/dl)	6.4 ± 1.6	6.4 ± 1.8	0.967
eGFR (ml/min/1.73 m^2^)	9.3 ± 5.7	7.0 ± 2.6	<0.0001
Creatinine (mg/dl)	8.6 ± 3.2	10.1 ± 5.9	0.008
Hemoglobin (g/dL)	9.9 ± 1.5	10.0 ± 1.1	0.298
Aspartate aminotransferase (U/L)	20.6 ± 13.2	18.6 ± 11.1	0.040
Alanine aminotransferase (U/L)	16.2 ± 10.7	16.5 ± 13.0	0.545
Alkaline phosphatase (U/L)	106.4 ± 57.9	99.2 ± 71.8	0.034
Potassium (mEq/l)	4.5 ± 0.7	4.6 ± 0.7	0.155
Calcium (mg/dL)	9.2 ± 0.9	9.1 ± 0.9	0.150
Phosphate (mg/dL)	4.7 ± 1.2	5.3 ± 1.2	<0.0001
Parathyroid hormone (pg/mL)	161.5(72.5-309.3)	272.2(144.4-573.4)	0.002
Albumin (g/dL)	3.7 ± 0.5	4.0 ± 0.3	<0.0001
White blood cell (10^9^/l)	6.930(5.790-8.440)	6.475(5.323-7.978)	0.923
Red blood cell (10 ^6^/μl)	3.356 ± 0.663	3.515 ± 1.538	0.260
Hs-CRP (mg/L)	7.3(1.7-15.9)	2.9(1.2-8.7)	0.001
Baseline retinol-binding protein 4 (ug/mL)	85.9 ± 21.4	91.2 ± 19.6	0.001
Longitudinal retinol-binding protein 4 (ug/mL)	84.5 ± 30.4	97.5 ± 24.6	<0.0001

Data are expressed as number (percentage), mean ± SD, or median (interquartile range). eGFR, estimated glomerular filtration rate; Hs-CRP, high-sensitivity C-reactive protein.

### Baseline biochemical and clinical characteristics of the patients stratified by baseline RBP4 concentration quartiles

The baseline clinical and biochemical characteristics of the patients categorized by baseline plasma RBP4 concentration quartiles are shown in **Table 2**. Patients in the lowest quartile were older, and had a shorter duration of hemodialysis, higher prevalence of diabetes mellitus and diabetic nephropathy as the etiology of ESKD, and lower incidence of hyperlipidemia and chronic glomerulonephritis as the etiology of ESKD. In addition, they had elevated fasting glucose, eGFR, and hs-CRP, along with lower diastolic blood pressure, total cholesterol, triglycerides, uric acid, creatinine, potassium, calcium, phosphate, parathyroid hormone, albumin, and red blood cells compared to the patients in the highest quartile (p for trend < 0.05).

**Table 2:** Baseline clinical and biochemical characteristics of patients stratified by quartiles of baseline retinol-binding protein 4 levels.

Variable	Q1 (<76.48 ug/mL)	Q2 (76.48-90.59 ug/mL)	Q3 (90.60-101.17 ug/mL)	Q4 (>101.17 ug/mL)	p for trend
Number	85	86	86	85	
Age (years)	69.3 ± 12.1	65.0 ± 13.8	61.9 ± 13.9	58.5 ± 11.9	<0.0001
Male (n, %)	50(58.8)	45(52.3)	50(58.1)	43(50.6)	0.437
Time on hemodialysis (years)	2.0(1.0-4.0)	4.0(1.0-7.0)	4.5(2.0-7.0)	5.0(3.0-7.5)	<0.0001
Comorbidities (n, %)					
Hypertension	49(57.7)	54(62.8)	60(69.8)	57(67.1)	0.223
Diabetes mellitus	71(83.5)	72(83.7)	55(64.0)	53(62.4)	0.0001
Hyperlipidemia	30(35.3)	29(33.7)	28(32.6)	46(54.1)	0.027
Etiology of ESRD (n, %)					
Diabetic nephropathy	58(68.2)	57(66.3)	39(45.4)	41(48.2)	0.001
Hypertensive nephropathy	10(11.8)	9(10.5)	15(17.4)	8(9.4)	0.382
Chronic glomerulonephritis	11(12.9)	16(18.6)	19(22.1)	28(32.9)	0.001
Others	6(7.1)	4(4.7)	13(15.1)	8(9.4)	0.207
Body mass index (kg/m^2^)	22.6 ± 4.4	23.4 ± 4.5	23.1 ± 4.0	23.0 ± 4.2	0.682
Systolic BP (mmHg)	143 ± 23	147 ± 23	147 ± 23	149 ± 19	0.138
Diastolic BP (mmHg)	69 ± 12	73 ± 12	71 ± 11	74 ± 11	0.011
Total cholesterol (mg/dl)	162.3 ± 58.9	159.9 ± 34.3	173.8 ± 32.2	177.0 ± 39.0	0.007
Triglyceride (mg/dl)	101.0(74.0-152.0)	105.0(71.8-151.8)	133.0(81.0-227.0)	129.0(83.0-194.0)	0.008
Fasting glucose (mg/dl)	159.5 ± 76.7	144.9 ± 78.1	150.2 ± 94.1	124.0 ± 61.2	0.010
Uric acid (mg/dl)	6.1 ± 1.9	6.3 ± 1.8	6.6 ± 1.4	6.8 ± 1.6	0.004
eGFR (ml/min/1.73 m^2^)	10.3 ± 6.8	8.0 ± 3.1	7.2 ± 2.9	6.3 ± 2.0	<0.0001
Creatinine (mg/dl)	7.6(5.6-9.1)	8.9(7.4-10.8)	9.3(7.8-11.4)	10.2(9.3-11.9)	0.014
Hemoglobin (g/dL)	9.7 ± 1.4	10.0 ± 1.2	10.1 ± 1.2	10.0 ± 1.3	0.094
Hematocrit (%)	29.5 ± 4.1	30.6 ± 3.7	30.5 ± 3.6	30.0 ± 3.8	0.439
Ferritin (ng/mL)	364.4(194.9-546.5)	325.8(163.1-493.3)	293.4(132.8-524.7)	397.3(237.3-512.6)	0.816
Transferrin saturation (%)	28.9(24.1-38.4)	31.0(23.0-36.1)	30.8(22.9-38.1)	29.8(25.2-35.9)	0.543
Potassium (meq/l)	4.2 ± 0.7	4.5 ± 0.6	4.7 ± 0.6	4.8 ± 0.7	<0.0001
Calcium (mg/dL)	8.9 ± 0.9	9.2 ± 0.9	9.1 ± 0.8	9.4 ± 0.9	0.002
Phosphate (mg/dL)	4.6 ± 1.2	4.9 ± 1.2	5.4 ± 1.4	5.3 ± 1.1	<0.0001
Parathyroid hormone (pg/mL)	177.8(86.2-278.7)	172.1(91.8-344.0)	263.5(97.8-567.3)	297.3(148.6-599.4)	0.002
Albumin (g/dL)	3.6 ± 0.5	3.9 ± 0.4	4.0 ± 0.3	4.0 ± 0.3	<0.0001
White blood cell (10^9^/l)	6.630(5.163-8.565)	6.685(5.370-8.743)	6.760(5.785-7.685)	6.550(5.340-8.448)	0.929
Red blood cell (10 ^6^/μl)	3.229 ± 0.510	3.396 ± 0.628	3.436 ± 0.530	3.743 ± 2.320	0.011
Hs-CRP (mg/L)	7.3(2.0-29.8)	2.8(1.3-11.0)	4.1(1.4-9.4)	4.2(1.0-10.2)	0.046

Data are expressed as number (percentage), mean ± SD, or median (interquartile range). ESRD, end stage renal disease; BP, blood pressure; eGFR, estimated glomerular filtration rate; AST, aspartate aminotransferase; ALT, alanine aminotransferase; Hs-CRP, high- sensitivity C-reactive protein.

### RBP4 levels and all-cause mortality

Associations between quartiles of baseline and longitudinal RBP4 levels and all-cause mortality are shown in **Table 3**. Following an average follow-up period of 4.3 years, a consistent decrease in mortality was observed across both baseline and longitudinal RBP4 quartiles. Importantly, as baseline RBP4 quartiles decreased, there was a proportional increase in the HR for mortality compared to Quartile (Q) 4 (HR: 1 for Q 4; HR: 1.48 for Q 3; HR: 1.89 for Q 2; and HR: 4.34 for Q 1; p-value for trend across decreasing quartiles < 0.0001). Similarly, with a decrease in longitudinal RBP4 quartile, there was a proportional increase in the HR for non-survivors relative to Q 4 (HR: 1 for Q 4; HR: 1.68 for Q 3; HR: 1.87 for Q 2; HR: 3.38 for Q 1; p-value for trend across decreasing quartiles < 0.0001).

**Table 3:** Hazard ratios (HRs) of all-cause mortality according to baseline and longitudinal plasma retinol-binding protein 4 quartiles followed for a mean 4.3 ± 3.6 years (N=342).

	All-cause mortality status			
Variables	Non-survivors	Survivors	HR (95%CI)	p-value
Baseline RBP4				
Q1 (<76.49 ug/mL)	35 (17.4%)	50 (35.5%)	4.34 (2.65-7.31)	<0.0001
Q2 (76.49-90.59 ug/mL)	50 (24.9%)	36 (25.5%)	1.89 (1.13-3.23)	0.016
Q3 (90.60-101.17 ug/mL)	55 (27.4%)	31 (22.0%)	1.48 (0.87-2.57)	0.149
Q4 (>101.17 ug/mL)	61 (30.4%)	24 (17.0%)	1.00 (reference)	
p for trend			<0.0001	
Longitudinal RBP4				
Q1 (<81.56 ug/mL)	31 (15.4%)	54 (38.3%)	3.38 (2.06-5.79)	<0.0001
Q2 (81.56-94.48 ug/mL)	50 (24.9%)	36 (25.5%)	1.87 (1.09-3.28)	0.022
Q3 (94.49-103.84 ug/mL)	54 (26.9%)	31 (22.0%)	1.68 (0.96-2.99)	0.068
Q4 (>103.84 ug/mL)	66 (32.8%)	20 (14.2%)	1.00 (reference)	
p for trend			<0.0001	

RBP, retinol-binding protein; HR, hazard ratio; CI, confidence interval.

### RBP4 levels and all-cause mortality stratified by sex

When patients were stratified by sex (**Supplementary Table 2**), univariate Cox regression analysis revealed a consistent inverse association between RBP4 levels and all-cause mortality across both baseline and longitudinal quartiles in males and females. In males, a decrease in baseline RBP4 quartiles was associated with a stepwise increase in the HR for all-cause mortality relative to Q 4: HR = 1.34 for Q 3, HR = 1.60 for Q 2, and HR = 4.20 for 1 (p for trend = 0.001). A similar trend was observed for longitudinal RBP4: HR = 1.71 (Q3), 1.70 (Q2), and 3.65 (Q1) versus Q 4 (p for trend < 0.0001). In females, decreasing baseline RBP4 quartiles were also associated with increasing all-cause mortality risk: HR = 1.59 (Q3), 2.21 (Q2), and 4.42 (Q1) compared to Q 4 (p for trend = 0.011). Likewise, lower longitudinal RBP4 levels were linked to higher all-cause mortality: HR = 1.60 (Q3), 1.92 (Q2), and 2.99 (Q1) relative to Q 4 (p for trend = 0.002).

### RBP4 levels and all-cause mortality stratified by diabetes status

When patients were stratified by diabetes status (**Supplementary Table 3**), univariate Cox regression analysis revealed a consistent inverse association between RBP4 levels and all-cause mortality across both baseline and longitudinal quartiles in both type 2 diabetes mellitus (T2DM) and non-T2DM groups. In patients with T2DM, decreasing baseline RBP4 quartiles were associated with progressively higher all-cause mortality risk: HR = 1.87 for Q 3, 1.49 for Q 2, and 4.01 for Q 1, compared to Q 4 (p for trend = 0.004). A similar trend was observed for longitudinal RBP4: HR = 1.27 (Q3), 1.24 (Q2), and 2.42 (Q1) versus Q 4 (p for trend = 0.001). Among patients without T2DM, lower baseline RBP4 quartiles were also significantly associated with increased all-cause mortality: HR = 0.83 (Q3), 3.16 (Q2), and 4.41 (Q1) compared to Q 4 (p for trend = 0.010). For longitudinal RBP4, the association was even more pronounced: HR = 2.37 (Q3), 4.06 (Q2), and 6.08 (Q1) relative to Q 4 (p for trend < 0.0001).

### RBP4 levels and all-cause mortality stratified by underlying cause of CKD

When patients were stratified by the underlying cause of CKD, univariate Cox regression analysis revealed a consistent inverse association between both baseline and longitudinal RBP4 quartiles and all-cause mortality across diabetic nephropathy (DN), glomerulonephritis (GN), and other etiologies (**Supplementary Table 4**). In patients with DN, lower baseline RBP4 quartiles were associated with significantly increased all-cause mortality risk compared to Q 4: HR = 1.38 (Q3), 1.34 (Q2), and 3.54 (Q1; p for trend < 0.0001). A similar trend was observed for longitudinal RBP4: HR = 1.11 (Q3), 1.15 (Q2), and 2.53 (Q1; p for trend < 0.0001). In the GN group, the association was even more pronounced. For baseline RBP4, the HRs were 0.96 (Q3), 3.22 (Q2), and 5.01 (Q1), compared to Q 4 (p for trend < 0.0001). Longitudinal RBP4 showed a striking increase in all-cause mortality risk in lower quartiles: HR = 4.71 (Q3), 4.59 (Q2), and 8.26 (Q1; p for trend < 0.0001). Among patients with other etiologies (including hypertensive nephrosclerosis, tubulointerstitial diseases, and polycystic kidney disease), the trend persisted. Baseline RBP4 quartiles were associated with increasing all-cause mortality risk: HR = 2.31 (Q3), 2.35 (Q2), and 5.14 (Q1; p for trend < 0.0001). Longitudinal RBP4 quartiles also showed a graded relationship: HR = 1.88 (Q3), 2.54 (Q2), and 2.98 (Q1; p for trend < 0.0001), although individual quartiles did not all reach statistical significance.

### Association between RBP4 levels with all-cause mortality

We used univariate and multivariate Cox regression models to explore associations between baseline and longitudinal RBP4 levels with all-cause mortality, as presented in **Table 4**. Individuals in Q 1 of baseline and longitudinal RBP4 levels had a higher risk of all-cause mortality compared to those in Q 4 across model 1, model 2, and model 3 (HR: 4.34, 95% CI 2.65-7.31, p < 0.0001; HR: 3.20, 95% CI 1.91-5.52, p < 0.0001; and HR: 2.22, 95% CI 1.16-4.31, p = 0.016, respectively, for baseline RBP4; HR: 3.38, 95% CI 2.06-5.79, p < 0.0001; HR: 2.50, 95% CI 1.49-4.36, p = 0.0004; and HR: 2.44, 95% CI 1.30-4.84, p = 0.005, respectively, for longitudinal RBP4). Conversely, the participants in Quartiles 2 and 3 of both baseline and longitudinal RBP4 levels did not have an increased risk of all-cause mortality compared to those in Quartile 4 across the three models, with the exception of participants in Quartile 2, who had an increased risk in model 1. Kaplan-Meier survival curves for all-cause mortality across baseline RBP4 quartiles are presented in **Supplementary Figure 2**. The participants in the lowest quartile (Quartile 1) had the poorest all-cause survival rate (p < 0.0001). In addition, ROC curve analysis demonstrated that baseline plasma RBP4 had an area under the curve (AUC) of 0.692 (p < 0.0001) for predicting all-cause mortality (**Supplementary Figure 3**). A threshold of <95.62 μg/mL was associated with a sensitivity of 70.9% and a specificity of 42.8%.

**Table 4:** Cox regression of the association of baseline and longitudinal plasma retinol-binding protein 4 with all-cause mortality.

	All-cause mortality		
	Model 1	Model 2	Model 3
Variables	HR (95%CI) p-value	HR (95%CI) p-value	HR (95%CI) p-value
Baseline RBP4			
Q1 (<76.49 ug/mL)	4.34 (2.65-7.31) <0.0001	3.20 (1.91-5.52) <0.0001	2.22 (1.16-4.31) 0.016
Q2 (76.49-90.59 ug/mL)	1.89 (1.13-3.23) 0.016	1.62 (0.96-2.79) 0.073	1.04 (0.56-1.95) 0.911
Q3 (90.60-101.17 ug/mL)	1.48 (0.87-2.57) 0.149	1.27 (0.74-2.21) 0.395	1.18 (0.64-2.18) 0.598
Q4 (>101.17 ug/mL)	Ref	Ref	Ref
Longitudinal RBP4			
Q1 (<81.56 ug/mL)	3.38 (2.06-5.79) <0.0001	2.50 (1.49-4.36) 0.0004	2.44 (1.30-4.84) 0.005
Q2 (81.56-94.48 ug/mL)	1.87 (1.09-3.28) 0.022	1.54 (0.89-2.73) 0.124	1.91 (0.97-3.91) 0.061
Q3 (94.49-103.84 ug/mL)	1.68 (0.96-2.99) 0.068	1.49 (0.85-2.67) 0.164	2.03 (1.01-4.22) 0.048
Q4 (>103.84 ug/mL)	Ref	Ref	Ref

Model 1: Univariate logistic regression analysis.

Model 2: Adjusted for age and gender.

Model 3: Adjusted for age, gender, body mass index, hypertension, diabetes mellitus, triglycerides, estimated glomerular filtration rate, hemoglobin, aspartate aminotransferase, alanine aminotransferase, and uric acid.

HR, hazard ratio.

### Evaluation of changes in RBP4 levels in the survivors and non-survivors

**Figure 1:**
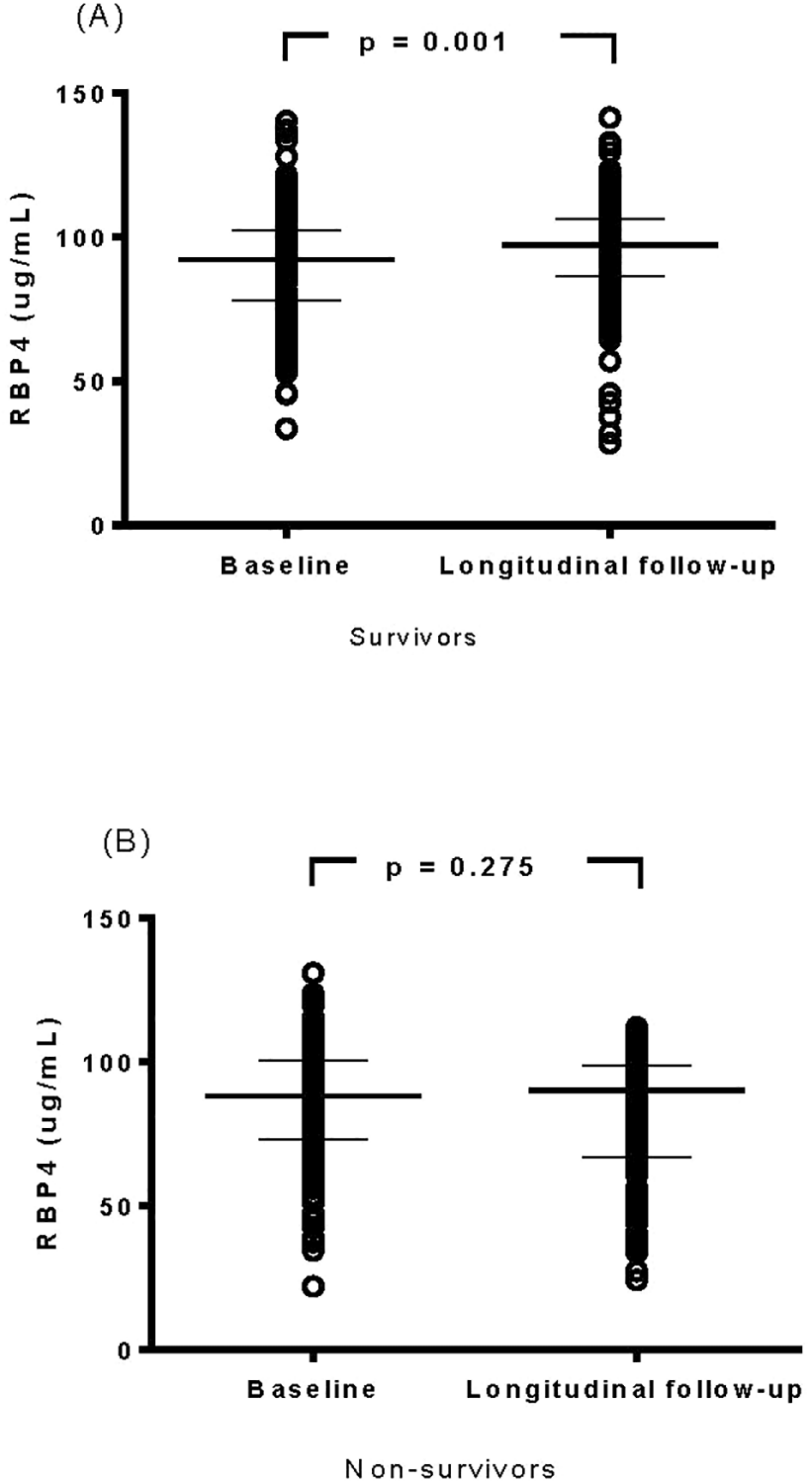
Evaluation of changes in cytokine levels of retinol-binding protein 4 in survivor **(A)** and non-survivor **(B)** groups.

The changes between baseline (initiation of hemodialysis) and longitudinal RBP4 levels (after 1 year of follow-up) were further analyzed in the survivor and non-survivor groups. Notably, RBP4 levels remained stable in the non-survivors (p = 0.275), whereas there was a significant increase after 1 year of follow-up in the survivors (Wilcoxon test, p = 0.001, **Figure 1**).

### Relationship between baseline plasma RBP4 levels and clinical and biochemical parameters

**Figure 2:**
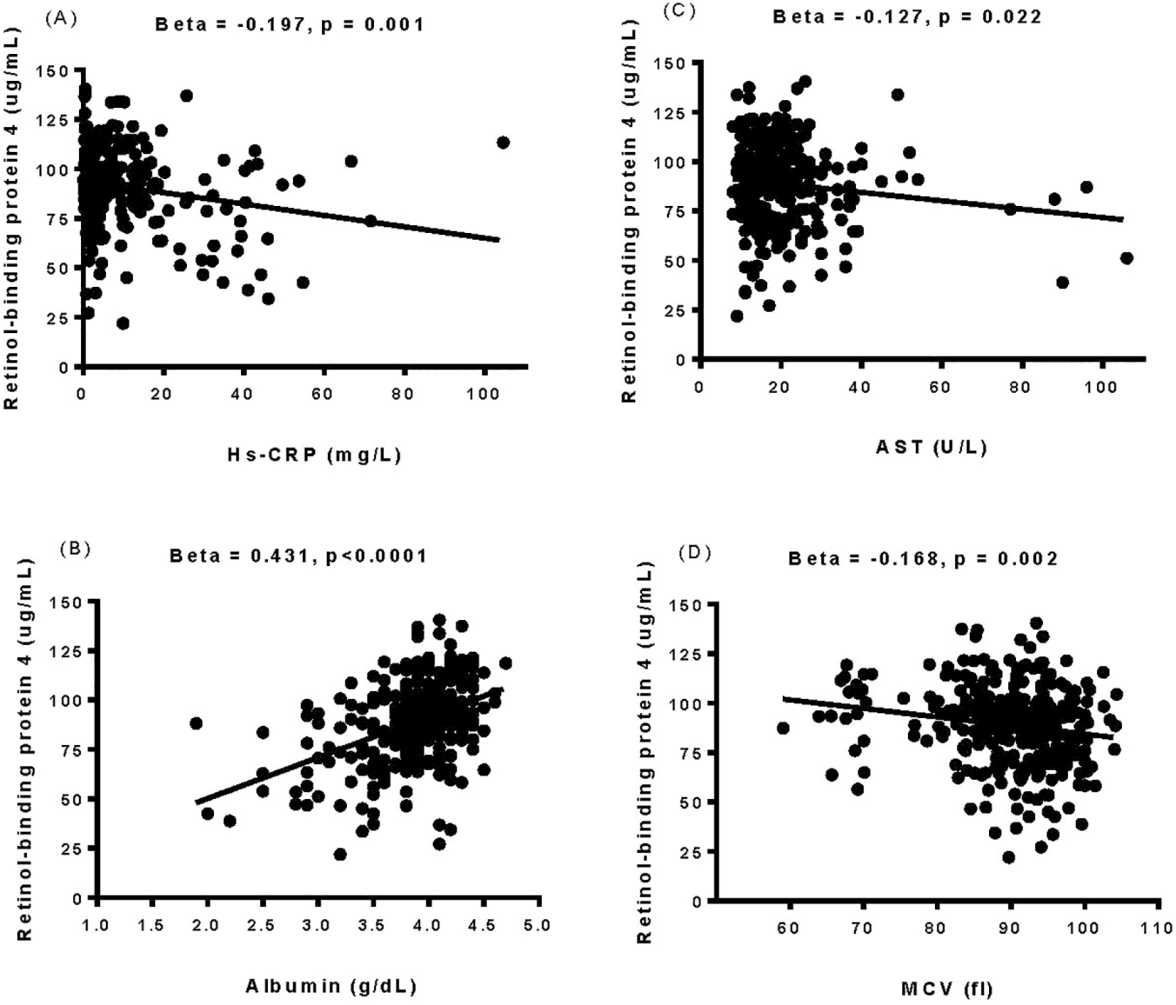
The relationship between baseline plasma retinol-binding protein 4 levels and the levels of high-sensitivity C-reactive protein **(A)**, albumin **(B)**, aspartate aminotransferase **(C)**, and mean corpuscular volume **(D)** was determined by simple linear regression analysis. Hs-CRP, high-sensitivity C-reactive protein; AST, aspartate aminotransferase; MCV, mean corpuscular volume.

Positive correlations were observed between baseline plasma RBP4 levels and duration of hemodialysis, systolic/diastolic blood pressures, total cholesterol, triglycerides, creatinine, uric acid, platelet count, hemoglobin, potassium, calcium, phosphate, parathyroid hormone, albumin, total protein, red blood cell count, and normalized protein catabolic rate. Conversely, negative correlations were identified between baseline plasma RBP4 levels and age, eGFR, mean corpuscular volume, AST, hs-CRP, and fasting glucose (as shown in **Supplementary Table 5**, **Figure 2**).

### Effect of baseline RBP4 on all-cause mortality

**Figure 3:**
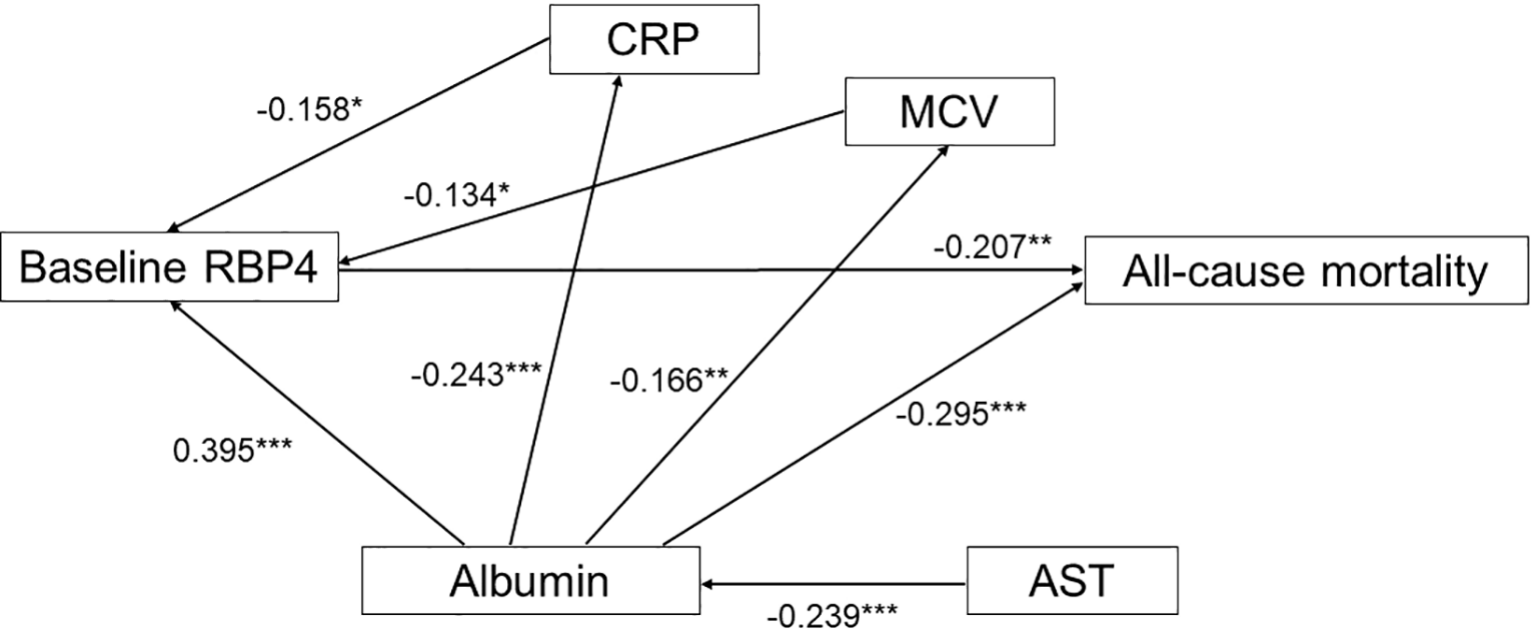
Estimated model of the relationship of baseline retinol-binding protein 4, high-sensitivity C-reactive protein, albumin, aspartate aminotransferase, and mean corpuscular volume, and their effect on all-cause mortality. Note: Coefficients are standardized path coefficients. Overall model fit, CFI = 0.996, SRMR = 0.035, RMSEA = 0.018. For tests of significance of individual paths, *p <0.05, **p <0.01, and ***p <0.001. RBP, retinol-binding protein; hs-CRP, high-sensitivity C-reactive protein; AST, aspartate aminotransferase; MCV, mean corpuscular volume.

Following Pearson’s correlation coefficient analysis shown in **Supplementary Table 5** and **Figure 2**, we constructed an SEM to evaluate the influence of RBP4 on all-cause mortality, as depicted in **Figure 3**. Baseline RBP4 (β = -0.207) and albumin (β = -0.295) demonstrated significant negative direct effects on all-cause mortality. In addition, hs-CRP (β = -0.158) and mean corpuscular volume (β = -0.134) had indirect effects on all-cause mortality through RBP4. Moreover, albumin (β = 0.395) indirectly influenced all-cause mortality through RBP4, and AST (β = -0.239) indirectly influenced all-cause mortality through albumin. The estimated model demonstrated a good fit, with a CFI of 0.996, an RMSEA of 0.018, and an SRMSR of 0.035 (**Figure 3**).

### Interaction effects of RBP4 with hs-CRP and albumin on all-cause mortality

To investigate potential interaction effects, Cox regression analyses were performed by including interaction terms between baseline RBP4 and hs-CRP, as well as baseline RBP4 and albumin (**Supplementary Table 6**). In Model 1 (univariate analysis), baseline RBP4 was significantly associated with reduced all-cause mortality (HR: 0.98, 95% CI: 0.97-0.98, p < 0.0001), while hs-CRP was positively associated with all-cause mortality (HR: 1.20, 95% CI: 1.06-1.37, p = 0.005). However, the interaction term between RBP4 and hs-CRP did not reach statistical significance (p = 0.281). After adjustment for age and gender in Model 2, the associations of baseline RBP4 (HR: 0.98, 95% CI: 0.97-0.99, p < 0.0001) and hs-CRP (HR: 1.15, 95% CI: 1.01-1.32, p = 0.039) with all-cause mortality remained significant, while the RBP4 × hs-CRP interaction remained non-significant (p = 0.104). In the fully adjusted Model 3, which included additional covariates such as body mass index, comorbidities, and laboratory parameters, baseline RBP4 (HR: 0.99, 95% CI: 0.98-1.00, p = 0.008) and hs-CRP (HR: 1.17, 95% CI: 1.00-1.38, p = 0.042) remained significantly associated with all-cause mortality, but the interaction term remained non-significant (p = 0.313). Similarly, in the models including albumin, baseline RBP4 and albumin were both significantly associated with reduced all-cause mortality across all models. In Model 3, baseline RBP4 had an HR of 0.99 (95% CI: 0.98-1.00, p = 0.023), and albumin had an HR of 0.38 (95% CI: 0.19-0.76, p = 0.007). However, the RBP4 × albumin interaction term was not statistically significant in any of the models (p = 0.752 in Model 3).

### Proposed mechanisms linking decreased RBP4 levels to all-cause mortality in maintenance hemodialysis patients

**Figure 4:**
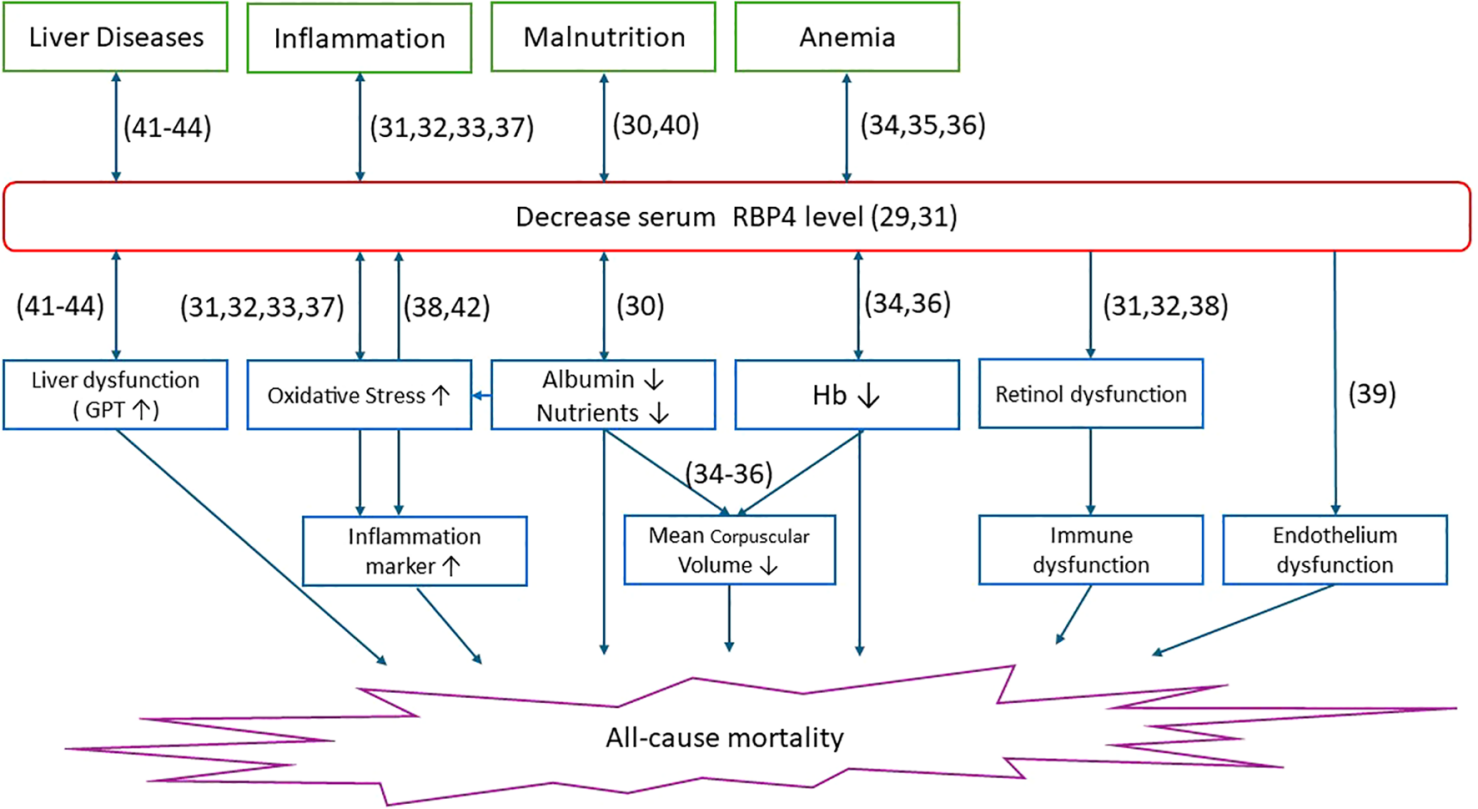
Multiple upstream clinical conditions including liver disease, inflammation, malnutrition, and anemia are associated with decreased circulating RBP4 levels. Reduced serum RBP4 may reflect underlying liver dysfunction, oxidative stress, poor nutritional status (e.g., hypoalbuminemia), and anemia (e.g., low hemoglobin), as well as potential disturbances in retinol transport. These conditions are linked to downstream pathophysiological mechanisms such as immune and endothelial dysfunction, ultimately contributing to increased risk of all-cause mortality. Numbers in parentheses indicate supporting reference numbers.

**Figure 4** summarizes the potential biological pathways through which decreased RBP4 levels may contribute to all-cause mortality in maintenance hemodialysis patients.

## Discussion

In this study, we investigated associations between baseline and longitudinal plasma RBP4 levels and all-cause mortality in patients undergoing maintenance hemodialysis. There were three main findings. First, the non-survivors had significantly lower baseline and longitudinal RBP4 levels compared to the survivors. In multivariate analysis, patients in the lowest quartiles (Quartile 1) of baseline and longitudinal RBP4 levels had HRs of 2.22 (95% CI: 1.16-4.31) and 2.44 (95% CI: 1.30-4.84) for all-cause mortality, respectively, compared to those in Quartile 4. Second, in contrast to the non-survivors, plasma RBP4 levels significantly increased during the 1-year follow-up period compared to baseline (initiating hemodialysis) in the survivors (p = 0.001). Third, SEM analysis confirmed causal relationships between baseline RBP4 levels, albumin, hs-CRP, AST, mean corpuscular volume, and all-cause mortality. In addition, our findings not only confirm previous associations between low RBP4 and mortality in dialysis patients, but also extend this relationship across key clinical subgroups.

Regarding the first major finding, 141 of the 342 patients (41.2%) died during the follow-up period. In analysis stratified by sex, 75 of the 188 male patients (39.9%) died, compared to 66 of the 154 female patients (42.9%). In Lee et al.’s study (24), 75,297 dialysis patients (58.4%) died during follow-up, including 36,834 of the 60,186 male patients (61.2%) and 34,649 of the 57,190 female patients (60.5%). Due to the high mortality rate of patients undergoing dialysis, it is crucial to explore the associated biomarkers. In the present study, we found highly elevated plasma concentrations of RBP4 in our patients compared to the general population, which is consistent with previous studies on RBP4 concentration in patients undergoing hemodialysis (13, 18). The mechanism by which physiological homeostatic regulation increases plasma levels of RBP4 in hemodialysis patients is unknown. Apo-RBP4 (RBP4 not bound to retinol) is considered to be a positive feedback signal from the periphery indicating the demand for retinol in target cells. Consequently, increased tissue uptake of retinol leads to elevated levels of apo-RBP4, stimulating the release of retinol in the liver. Decreased kidney function results in reduced filtration and degradation of RBP4, leading to a subsequent increase in serum concentration of apo-RBP4, possibly triggering enhanced retinol release from the liver (8, 25). In the present study, we observed significantly lower baseline and longitudinal RBP4 levels in the non-survivors compared to the survivors (**Table 1**). Nevertheless, these low RBP4 plasma concentrations were still higher than the recommended levels for healthy individuals. Furthermore, after multivariate Cox regression analysis, individuals in the lowest quartiles (Q 1) of baseline and longitudinal RBP4 levels were associated with all-cause mortality (**Table 4**). Consistent with our findings, a prior investigation reported a robust correlation between lower concentrations of RBP4 with sudden cardiac death and all-cause mortality among diabetic hemodialysis patients (18). Conversely, Ingelsson et al. reported that in models adjusted for multiple variables, RBP4 exhibited a positive association with metabolic syndrome and previous cerebrovascular events, suggesting its potential role as an indicator of metabolic complications, and conceivably, atherosclerosis and overt CVD (26). Kalousova et al. demonstrated the significance of RBP4 levels as a biochemical predictor for both overall and cardiovascular mortality (27). In addition, Cabré et al. observed a positive association between plasma RBP4 concentration and serum creatinine level along with an inverse relationship with eGFR, suggesting that plasma RBP4 concentration may be a potential biomarker for nephropathy and CVD in individuals with type 2 diabetes (15). Consequently, the precise role of RBP4 remains elusive in patients undergoing maintenance hemodialysis. Our findings provide further insights by identifying low RBP4 levels as an important risk factor for all-cause mortality within this patient population.

To further assess the consistency of this association across clinically relevant subgroups, we conducted stratified analyses based on sex, diabetes status, and underlying cause of CKD. Consistent inverse associations between RBP4 levels and all-cause mortality were observed across all subgroups. In both males and females, decreasing quartiles of baseline and longitudinal RBP4 levels were associated with progressively higher mortality risks, with the lowest quartile exhibiting the highest hazard ratios (p for trend = 0.001 and <0.0001 for males, and 0.011 and 0.002 for females, respectively; **Supplementary Table 2**). Similarly, in patients with and without T2DM, lower RBP4 levels were significantly associated with increased mortality (p for trend = 0.004 and 0.001 in T2DM; 0.010 and <0.0001 in non-T2DM; **Supplementary Table 3**). Stratification by CKD etiology revealed a consistent pattern across patients with diabetic nephropathy, glomerulonephritis, and other causes, with the strongest associations observed in the glomerulonephritis group (**Supplementary Table 4**). Taken together, these findings suggest that low plasma RBP4 may serve as a broadly applicable prognostic biomarker in maintenance hemodialysis patients, regardless of sex, diabetes status, or underlying renal pathology.

To further evaluate the predictive utility of RBP4, ROC curve analysis was performed. The results demonstrated that baseline plasma RBP4 had an AUC of 0.692 (p < 0.0001) for predicting all-cause mortality (**Supplementary Figure 3**), indicating a fair discriminatory ability. A threshold of <95.62 μg/mL yielded a sensitivity of 70.9% and a specificity of 42.8%, suggesting that low RBP4 levels may aid in identifying high-risk individuals, albeit with modest specificity. While not sufficient as a standalone diagnostic tool, RBP4 may have clinical value when used in conjunction with other established risk markers in this population.

Regarding the second important finding of this study, we used Kaplan-Meier survival curves to illustrate all-cause mortality across quartiles of baseline RBP4 levels, and found that patients in the lowest quartile (Quartile 1) had the poorest all-cause survival rate (**Supplementary Figure 2**). In addition, compared to baseline, plasma levels of RBP4 were significantly higher in the survivors during the 1-year follow-up period, whereas this increase was not observed in the non-survivors (**Figure 1**). These dynamic fluctuations in RBP4 levels suggest that serial monitoring of plasma RBP4 levels may be of predictive value for all-cause mortality in maintenance hemodialysis patients.

This study is the first to investigate the causal relationship between RBP4 and all-cause mortality among patients undergoing maintenance hemodialysis. Our SEM analysis showed significant negative direct effects of baseline RBP4 and albumin levels on the incidence of all-cause mortality. Similar findings have been reported in various clinical settings. For example, Chen et al. reported that in patients in an intensive care unit, the survivors had elevated baseline RBP4 levels compared to their counterparts, suggesting the potential utility of baseline RBP4 as a prognostic indicator for short-term mortality (28). In addition, Tang et al. reported that early assessments of albumin level not only reflected the nutritional and chronic inflammation status of hemodialysis patients, but also that they had prognostic value, thereby offering insights into the clinical management of such patients (29). In the present study, we found that hs-CRP and mean corpuscular volume exerted indirect effects on all-cause mortality through RBP4. A prior investigation showed a significant correlation between elevated inflammatory markers, particularly CRP, and lower plasma RBP4 levels in critically ill COVID-19 patients. The authors concluded that plasma levels of RBP4 are significantly reduced during acute inflammation in critically ill COVID-19 patients (30). In the present study, we observed a similar trend, with higher levels of hs-CRP and lower levels of RBP4 in the non-survivors compared to the survivors (**Table 1**). This finding raises the possibility that lower RBP4 levels among the non-survivors may have been due to heightened inflammatory responses, potentially triggered by factors associated with chronic hemodialysis such as grafts, fistulas, dialysis membranes, or infection sites. These reactions are often accompanied by elevated levels of inflammatory markers including serum CRP, cytokines, and notably, hs-CRP (31). Elevated serum CRP levels may contribute to the development of cardiovascular complications and mortality in hemodialysis patients (32). In addition, the association between high mean corpuscular volume and low RBP4 levels with all-cause mortality in hemodialysis patients likely involves complex interactions among anemia (33–35), inflammation (36), oxidative stress (37), endothelial dysfunction (38), and disrupted vitamin A metabolism (30). Further research is needed to elucidate the specific mechanistic pathways and identify potential therapeutic targets to improve outcomes in this patient population. Moreover, we also found that the patients in the lowest quartile of RBP4 had a lower level of albumin compared to those in the highest quartile. Our SEM analysis showed that albumin had a positive indirect effect on all-cause mortality mediated through RBP4. These observations may be explained by factors such as malnutrition, inflammation, oxidative stress, CVD, and liver dysfunction, all of which are intricately associated with the consequences of diminished albumin and RBP4 levels (18, 39). In addition, we found that AST indirectly influenced all-cause mortality through albumin. Elevated AST levels, a marker of hepatic dysfunction, can directly inhibit albumin synthesis via mechanisms involving inflammation (40), oxidative stress (41), and uremic toxins (42, 43). This impairment in albumin synthesis may contribute to increased all-cause mortality among hemodialysis patients. To further visualize and integrate these findings, **Figure 4** illustrates the proposed biological pathways through which decreased RBP4 levels may contribute to all-cause mortality in maintenance hemodialysis patients. RBP4, a hepatically synthesized protein, plays a central role in retinol transport and is influenced by multiple pathophysiological conditions common in end-stage renal disease. First, malnutrition, as indicated by low serum albumin, is associated with reduced hepatic protein synthesis and has been linked to lower RBP4 levels (29, 39). Our SEM analysis revealed that albumin had a positive indirect effect on all-cause mortality mediated through RBP4. Second, inflammation, reflected by elevated hs-CRP levels, is known to suppress RBP4 expression and promote protein catabolism (30–32, 36). Third, oxidative stress, often elevated in hemodialysis patients, may impair cellular homeostasis and antioxidant defense, indirectly contributing to RBP4 depletion (37, 41). Fourth, liver dysfunction, represented by elevated AST levels, may impair the synthesis of both RBP4 and albumin, further exacerbating the decline in nutritional and inflammatory balance (40–43). These interconnected mechanisms may lead to decreased RBP4 levels, which, as demonstrated in our multivariate analysis and SEM, are significantly associated with increased all-cause mortality. Understanding these mechanisms provides insight into the clinical significance of RBP4 as a potential biomarker and therapeutic target in the management of hemodialysis patients.

To further investigate whether the associations between RBP4 and mortality were modified by systemic inflammation or nutritional status, interaction analyses were conducted using Cox regression models including interaction terms between baseline RBP4 and hs-CRP, as well as RBP4 and albumin (**Supplementary Table 6**). In all models, baseline RBP4 remained significantly associated with reduced all-cause mortality, and hs-CRP and albumin retained their respective associations with increased and decreased mortality risk. However, none of the interaction terms reached statistical significance across the univariate, age- and sex-adjusted, or fully adjusted models. These findings suggest that the prognostic value of RBP4 is largely independent of hs-CRP and albumin levels, reinforcing its potential role as an additive, rather than synergistic, risk marker in the clinical setting.

## Limitations

There were several limitations to this study. First, it was conducted at a single center, which may limit the extrapolation of our results to broader populations. Multi-center studies are warranted to validate our findings across diverse patient populations. Second, the sample size of the study cohort may have limited the generalizability of our findings. A larger sample size would provide more robust evidence of the associations observed. Third, Factors such as vitamin A supplementation, liver function, and the use of anti-inflammatory or immunosuppressive medications are known to affect circulating RBP4 levels. In the present study, all dialysis patients were clinically stable, without acute symptoms or comorbidities unrelated to kidney disease. Patients with chronic progressive conditions such as heart failure, malignancy, or active systemic lupus erythematosus as well as those using immunosuppressive agents or developing acute infections within three months of initiating hemodialysis were excluded. Therefore, none of the participants were taking vitamin A supplements, anti-inflammatory drugs, immunosuppressants, or other related medications. Moreover, we have included liver function parameters (aspartate aminotransferase and alanine aminotransferase) as additional covariates in the multivariable models to account for their potential impact on plasma RBP4 levels. Based on these considerations, we believe that the influence of confounding factors on our findings is likely to be minimal. However, we acknowledge that certain unmeasured factors such as vitamin A or zinc status, circulating IL-6 levels, and subjective nutritional scores were not available in this dataset and may have contributed to residual confounding. Fourth, selection bias was possible when assessing the association between changes in RBP4 levels and all-cause mortality. This is because we included only patients with at least two RBP4 measurements, and those with lower RBP4 levels may have died before subsequent measurements were conducted. Fifth, we use of only two timepoints limits our ability to assess temporal trends and dynamic changes in RBP4 levels. Future studies with more frequent longitudinal measurements are warranted to better characterize the temporal relationship between RBP4 and clinical outcomes. Finally, we did not use competing risk models (e.g., Fine and Gray) to account for specific causes of death, such as cardiovascular or infection-related events, due to incomplete data on cause-specific mortality. Our analysis was therefore limited to all-cause mortality. Future studies with more detailed death classifications may consider applying competing risk approaches to better delineate the prognostic role of RBP4.

In conclusion, our results revealed associations between low baseline and longitudinal plasma RBP4 concentrations and all-cause mortality among maintenance hemodialysis patients. This suggests that decreased plasma RBP4 levels may contribute to the pathogenesis of all-cause mortality in this patient population.

## Data Availability

The original contributions presented in the study are included in the article/**Supplementary Material**. Further inquiries can be directed to the corresponding author.
