# Supplementary material for: Association of baseline and longitudinal plasma retinol-binding protein 4 with all-cause mortality in maintenance hemodialysis patients
Source: Front Endocrinol (Lausanne). 2025 Aug 13;16:1434757. doi: 10.3389/fendo.2025.1434757 (PMC12381377; doi:10.3389/fendo.2025.1434757)
Supplement: Supplementary file 1 [file DataSheet1.doc]

**SUPPLEMENTARY DATA**

**Association of baseline and longitudinal plasma**

**retinol-binding protein 4 with all-cause mortality in maintenance hemodialysis patients**

**Contents**

Supplementary Figure 1. Circulating concentrations of baseline retinol-binding protein 4 in patients with hemodialysis and healthy controls. Bars represent the median (interquartile range). Differences between the groups were analyzed using Student’s *t* test..…….…..3

Supplementary Table 1. Baseline characteristics of the study population………………..4

Supplementary Table 2. Hazard ratios (HRs) of all-cause mortality according to baseline and longitudinal plasma retinol-binding protein 4 quartiles, stratified by sex (mean follow-up: 4.3 ± 3.6 years) ………………..………………..………………..………………...5

Supplementary Table 3. Hazard ratios (HRs) of all-cause mortality according to baseline and longitudinal plasma retinol-binding protein 4 quartiles, stratified by diabetes status (mean follow-up: 4.3 ± 3.6 years) …..………………..………………..……………………..6

Supplementary Table 4. Hazard ratios (HRs) of all-cause mortality according to quartiles of baseline and longitudinal plasma retinol-binding protein 4, stratified by the underlying cause of chronic kidney disease (mean follow-up: 4.3 ± 3.6 years) …..………………….8

Supplementary Figure 2. Kaplan-Meier survival curves were plotted to assess all-cause mortality across quartiles of retinol-binding protein 4 levels at baseline. The p-value indicates the significance of the log-rank test conducted across quartiles…...................9

Supplementary Figure 3. Receiver operator characteristics (ROC) curve of baseline retinol-binding protein 4 for the detection of all-cause mortality. A threshold value of <95.62 ug/mL is associated with all-cause mortality with a sensitivity of 70.9% and specificity of 42.8%…..………………..……………………………………………………….10

Supplementary Table 5. Relationship between baseline plasma retinol-binding protein 4 levels and clinical and biochemical parameters…..........................................................11

Supplementary Table 6. Cox regression analysis of all-cause mortality including interaction terms: baseline RBP4 × hs-CRP and baseline RBP4 × albumin………………………….12

Supplementary Figure 1. Circulating concentrations of baseline retinol-binding protein 4 in patients with hemodialysis and healthy controls. Bars represent the median (interquartile range). Differences between the groups were analyzed using Student’s *t* test.

Supplementary Table 1. Baseline characteristics of the study population

| Clinical characteristics | Total (n=342) |
| --- | --- |
| Retinol-binding protein 4 (ug/mL, mean ± SD) | 89.0±20.5 |
| Age (years, mean ± SD) | 63.7±13.5 |
| Male, n (%) | 188(55.0) |
| Time on hemodialysis (years, median (interquartile range)) | 4.0(1.0-6.0) |
| Comorbidities |  |
| Hypertension (n, %) | 220(64.3) |
| Diabetes mellitus (n, %) | 251(73.4) |
| Hyperlipidemia (n, %) | 133(38.9) |
| Etiology of end stage renal disease |  |
| Diabetic nephropathy, n (%) | 195(57.0) |
| Hypertensive nephropathy, n (%) | 42(12.3) |
| Chronic glomerulonephritis, n (%) | 74(21.6) |
| Others, n (%) | 31(9.1) |
| Body mass index (kg/m^2^, mean ± SD) | 23.1±4.3 |
| Systolic pressure (mmHg, mean ± SD) | 147±22 |
| Diastolic pressure (mmHg, mean ± SD) | 72±11 |
| Total cholesterol (mg/dl, mean ± SD) | 168.2±42.8 |
| Triglyceride (mg/dl, median (interquartile range)) | 117.0(77.0-169.0) |
| Fasting glucose (mg/dl, mean ± SD) | 144.7±79.1 |
| Uric acid (mg/dl, mean ± SD) | 6.4±1.7 |
| Estimated glomerular filtration rate (ml/min/1.73 m^2^, mean ± SD) | 7.9±4.3 |
| Creatinine (mg/dl, mean ± SD) | 9.5±5.0 |
| Hemoglobin (g/dL, mean ± SD) | 9.9±1.3 |
| Hematocrit (%, mean ± SD) | 30.1±3.8 |
| Ferritin (ng/mL, median (interquartile range)) | 357.0(168.1-524.3) |
| Transferrin saturation (%, mean ± SD) | 33.0±21.2 |
| Potassium (meq/l, mean ± SD) | 4.5±0.7 |
| Calcium (mg/dL, mean ± SD) | 9.2±0.9 |
| Phosphate (mg/dL, mean ± SD) | 5.0±1.3 |
| Parathyroid hormone (pg/mL, median (interquartile range)) | 213.7(97.0-447.1) |
| Albumin (g/dL, mean ± SD) | 3.9±0.4 |
| White blood cell (10^9^/l, mean ± SD) | 7.262±4.291 |
| Red blood cell (10 6/μl, mean ± SD) | 3.450±1.254 |
| High-sensitivity C-reactive protein (mg/L, median (interquartile range) | 4.0(1.4-12.3) |
| Kt/V (median (interquartile range)) | 1.3(1.1-1.5) |
| Urea reduction ratio (%, mean ± SD) | 0.72±0.06 |
| Normalized protein catabolic rate (mean ± SD) | 1.19±0.32 |

Supplementary Table 2. Hazard ratios (HRs) of all-cause mortality according to baseline and longitudinal plasma retinol-binding protein 4 quartiles, stratified by sex (mean follow-up: 4.3 ± 3.6 years)

|  | All-cause mortality status | | | |  | |
| --- | --- | --- | --- | --- | --- | --- |
| Variables | Non-survivors | Survivors | HR (95%CI) | p-value | |  |
| **Male (n=188)** |  |  |  |  | |  |
| **Baseline RBP4** |  |  |  |  | |  |
| Q1 (<76.49 ug/mL) | 29 (38.7%) | 21 (18.6%) | 4.20 (2.13-8.90) | <0.0001 | |  |
| Q2 (76.49-90.59 ug/mL) | 18 (24.0%) | 27 (23.9%) | 1.60 (0.77-3.50) | 0.214 | |  |
| Q3 (90.60-101.17 ug/mL) | 17 (22.7%) | 33 (29.2%) | 1.34 (0.64-2.96) | 0.442 | |  |
| Q4 (>101.17 ug/mL) | 11 (14.7%) | 32 (28.3%) | 1.00 (reference) |  | |  |
| p for trend |  |  | 0.001 |  | |  |
| **Longitudinal RBP4** |  |  |  |  | |  |
| Q1 (<81.56 ug/mL) | 31 (41.3%) | 15 (13.3%) | 3.65 (1.89-7.60) | <0.0001 | |  |
| Q2 (81.56-94.48 ug/mL) | 18 (24.0%) | 28 (24.8%) | 1.70 (0.82-3.72) | 0.158 | |  |
| Q3 (94.49-103.84 ug/mL) | 15 (20.0%) | 33 (29.2%) | 1.71 (0.79-3.83) | 0.172 | |  |
| Q4 (>103.84 ug/mL) | 11 (14.7%) | 37 (32.7%) | 1.00 (reference) |  | |  |
| p for trend |  |  | <0.0001 |  | |  |
| **Female (n=154)** |  |  |  |  | |  |
| **Baseline RBP4** |  |  |  |  | |  |
| Q1 (<76.49 ug/mL) | 21 (31.8%) | 14 (15.9%) | 4.42 (2.17-9.42) | <0.0001 | |  |
| Q2 (76.49-90.59 ug/mL) | 18 (27.3%) | 23 (26.1%) | 2.21 (1.07-4.75) | 0.032 | |  |
| Q3 (90.60-101.17 ug/mL) | 14 (21.2%) | 22 (25.0%) | 1.59 (0.74-3.51) | 0.236 | |  |
| Q4 (>101.17 ug/mL) | 13 (19.7%) | 29 (33.0%) | 1.00 (reference) |  | |  |
| p for trend |  |  | 0.011 |  | |  |
| **Longitudinal RBP4** |  |  |  |  | |  |
| Q1 (<81.56 ug/mL) | 23 (34.9%) | 16 (18.2%) | 2.99 (1.43-6.84) | 0.003 | |  |
| Q2 (81.56-94.48 ug/mL) | 18 (27.3%) | 22 (25.0%) | 1.92 (0.88-4.51) | 0.105 | |  |
| Q3 (94.49-103.84 ug/mL) | 16 (24.2%) | 21 (23.8%) | 1.60 (0.72-3.78) | 0.251 | |  |
| Q4 (>103.84 ug/mL) | 9 (13.6%) | 29 (33.0%) | 1.00 (reference) |  | |  |
| p for trend |  |  | 0.002 |  | |  |

RBP, retinol-binding protein; HR, hazard ratio; CI, confidence interval.

Supplementary Table 3. Hazard ratios (HRs) of all-cause mortality according to baseline and longitudinal plasma retinol-binding protein 4 quartiles, stratified by diabetes status (mean follow-up: 4.3 ± 3.6 years)

|  | All-cause mortality status | | | |  | |
| --- | --- | --- | --- | --- | --- | --- |
| Variables | Non-survivors | Survivors | HR (95%CI) | p-value | |  |
| **T2DM (n=232)** |  |  |  |  | |  |
| **Baseline RBP4** |  |  |  |  | |  |
| Q1 (<76.49 ug/mL) | 39 (36.8%) | 25 (19.8%) | 4.01 (2.24-7.58) | <0.0001 | |  |
| Q2 (76.49-90.59 ug/mL) | 27 (25.5%) | 40 (31.8%) | 1.49 (0.80-2.86) | 0.211 | |  |
| Q3 (90.60-101.17 ug/mL) | 25 (23.6%) | 26 (20.6%) | 1.87 (0.99-3.63) | 0.051 | |  |
| Q4 (>101.17 ug/mL) | 15 (14.2%) | 35 (27.8%) | 1.00 (reference) |  | |  |
| p for trend |  |  | 0.004 |  | |  |
| **Longitudinal RBP4** |  |  |  |  | |  |
| Q1 (<81.56 ug/mL) | 41 (38.7%) | 22 (17.4%) | 2.42 (1.39-4.44) | 0.002 | |  |
| Q2 (81.56-94.48 ug/mL) | 24 (22.6%) | 35 (27.8%) | 1.24 (0.66-2.37) | 0.511 | |  |
| Q3 (94.49-103.84 ug/mL) | 25 (23.6%) | 35 (27.8%) | 1.27 (0.68-2.42) | 0.459 | |  |
| Q4 (>103.84 ug/mL) | 16 (15.1%) | 34 (27.0%) | 1.00 (reference) |  | |  |
| p for trend |  |  | 0.001 |  | |  |
| **Non-T2DM (n=110)** |  |  |  |  | |  |
| **Baseline RBP4** |  |  |  |  | |  |
| Q1 (<76.49 ug/mL) | 11 (31.4%) | 10 (13.3%) | 4.41 (1.73-11.85) | 0.002 | |  |
| Q2 (76.49-90.59 ug/mL) | 9 (25.7%) | 10 (13.3%) | 3.16 (1.18-8.67) | 0.022 | |  |
| Q3 (90.60-101.17 ug/mL) | 6 (17.2%) | 29 (38.7%) | 0.83 (0.27-2.41) | 0.736 | |  |
| Q4 (>101.17 ug/mL) | 9 (25.7%) | 26 (34.7%) | 1.00 (reference) |  | |  |
| p for trend |  |  | 0.010 |  | |  |
| **Longitudinal RBP4** |  |  |  |  | |  |
| Q1 (<81.56 ug/mL) | 13 (37.1%) | 9 (12.0%) | 6.08 (2.14-21.67) | 0.001 | |  |
| Q2 (81.56-94.48 ug/mL) | 12 (34.3%) | 15 (20.0%) | 4.06 (1.41-14.53) | 0.009 | |  |
| Q3 (94.49-103.84 ug/mL) | 6 (17.2%) | 19 (25.3%) | 2.37 (0.67-9.33) | 0.180 | |  |
| Q4 (>103.84 ug/mL) | 4 (11.4%) | 32 (42.7%) | 1.00 (reference) |  | |  |
| p for trend |  |  | <0.0001 |  | |  |

T2DM, type 2 diabetes mellitus; RBP, retinol-binding protein; HR, hazard ratio; CI, confidence interval.

Supplementary Table 4. Hazard ratios (HRs) of all-cause mortality according to quartiles of baseline and longitudinal plasma retinol-binding protein 4, stratified by the underlying cause of chronic kidney disease (mean follow-up: 4.3 ± 3.6 years)

|  | All-cause mortality status | | | |  | |
| --- | --- | --- | --- | --- | --- | --- |
| Variables | Non-survivors | Survivors | HR (95%CI) | p-value | |  |
| **DN (n=195)** |  |  |  |  | |  |
| **Baseline RBP4** |  |  |  |  | |  |
| Q1 (<76.49 ug/mL) | 34 (40.0%) | 24 (21.8%) | 3.54 (1.89-7.01) | <0.0001 | |  |
| Q2 (76.49-90.59 ug/mL) | 22 (25.9%) | 35 (31.8%) | 1.34 (0.68-2.73) | 0.404 | |  |
| Q3 (90.60-101.17 ug/mL) | 16 (18.8%) | 23 (20.9%) | 1.38 (0.66-2.92) | 0.389 | |  |
| Q4 (>101.17 ug/mL) | 13 (15.3%) | 28 (25.5%) | 1.00 (reference) |  | |  |
| p for trend |  |  | <0.0001 |  | |  |
| **Longitudinal RBP4** |  |  |  |  | |  |
| Q1 (<81.56 ug/mL) | 35 (41.2%) | 19 (17.3%) | 2.53 (1.39-4.86) | 0.002 | |  |
| Q2 (81.56-94.48 ug/mL) | 18 (21.2%) | 28 (25.4%) | 1.15 (0.57-2.36) | 0.693 | |  |
| Q3 (94.49-103.84 ug/mL) | 18 (21.2%) | 32 (29.1%) | 1.11 (0.56-2.28) | 0.764 | |  |
| Q4 (>103.84 ug/mL) | 14 (16.4%) | 31 (28.2%) | 1.00 (reference) |  | |  |
| p for trend |  |  | <0.0001 |  | |  |
| **GN (n=74)** |  |  |  |  | |  |
| **Baseline RBP4** |  |  |  |  | |  |
| Q1 (<76.49 ug/mL) | 8 (27.6%) | 3 (6.7%) | 5.01 (1.76-14.78) | 0.003 | |  |
| Q2 (76.49-90.59 ug/mL) | 10 (34.5%) | 6 (13.3%) | 3.22 (1.21-9.11) | 0.019 | |  |
| Q3 (90.60-101.17 ug/mL) | 4 (13.8%) | 15 (33.3%) | 0.96 (0.25-3.17) | 0.941 | |  |
| Q4 (>101.17 ug/mL) | 7 (24.1%) | 21 (46.7%) | 1.00 (reference) |  | |  |
| p for trend |  |  | <0.0001 |  | |  |
| **Longitudinal RBP4** |  |  |  |  | |  |
| Q1 (<81.56 ug/mL) | 12 (41.4%) | 5 (11.1%) | 8.26 (2.24-53.30) | 0.001 | |  |
| Q2 (81.56-94.48 ug/mL) | 7 (24.1%) | 9 (20.0%) | 4.59 (1.11-30.80) | 0.035 | |  |
| Q3 (94.49-103.84 ug/mL) | 8 (27.6%) | 13 (28.9%) | 4.71 (1.17-31.26) | 0.028 | |  |
| Q4 (>103.84 ug/mL) | 2 (6.9%) | 18 (40.0%) | 1.00 (reference) |  | |  |
| p for trend |  |  | <0.0001 |  | |  |
| **Others* (n=73)** |  |  |  |  | |  |
| **Baseline RBP4** |  |  |  |  | |  |
| Q1 (<76.49 ug/mL) | 8 (29.6%) | 8 (17.4%) | 5.14 (1.44-24.1) | 0.011 | |  |
| Q2 (76.49-90.59 ug/mL) | 4 (14.8%) | 9 (19.6%) | 2.35 (0.52-12.0) | 0.262 | |  |
| Q3 (90.60-101.17 ug/mL) | 11 (40.8%) | 17 (36.9%) | 2.31 (0.72-10.25) | 0.169 | |  |
| Q4 (>101.17 ug/mL) | 4 (14.8%) | 12 (26.1%) | 1.00 (reference) |  | |  |
| p for trend |  |  | <0.0001 |  | |  |
| **Longitudinal RBP4** |  |  |  |  | |  |

Supplementary Table 4. Continue

|  | All-cause mortality status | | | |  | |
| --- | --- | --- | --- | --- | --- | --- |
| Variables | Non-survivors | Survivors | HR (95%CI) | p-value | |  |
| Q1 (<76.49 ug/mL) | 7 (25.9%) | 7 (15.2%) | 2.98 (0.90-11.39) | 0.075 | |  |
| Q2 (76.49-90.59 ug/mL) | 11 (40.8%) | 13 (28.2%) | 2.54 (0.85-9.28) | 0.098 | |  |
| Q3 (90.60-101.17 ug/mL) | 5 (18.5%) | 9 (19.6%) | 1.88 (0.50-7.61) | 0.345 | |  |
| Q4 (>101.17 ug/mL) | 4 (14.8%) | 17 (37.0%) | 1.00 (reference) |  | |  |
| p for trend |  |  | <0.0001 |  | |  |

DN, diabetic nephropathy; GN, glomerulonephritis; RBP, retinol-binding protein; HR, hazard ratio; CI, confidence interval.* Others included hypertensive nephrosclerosis, tubulointerstitial diseases, and polycystic kidney disease.


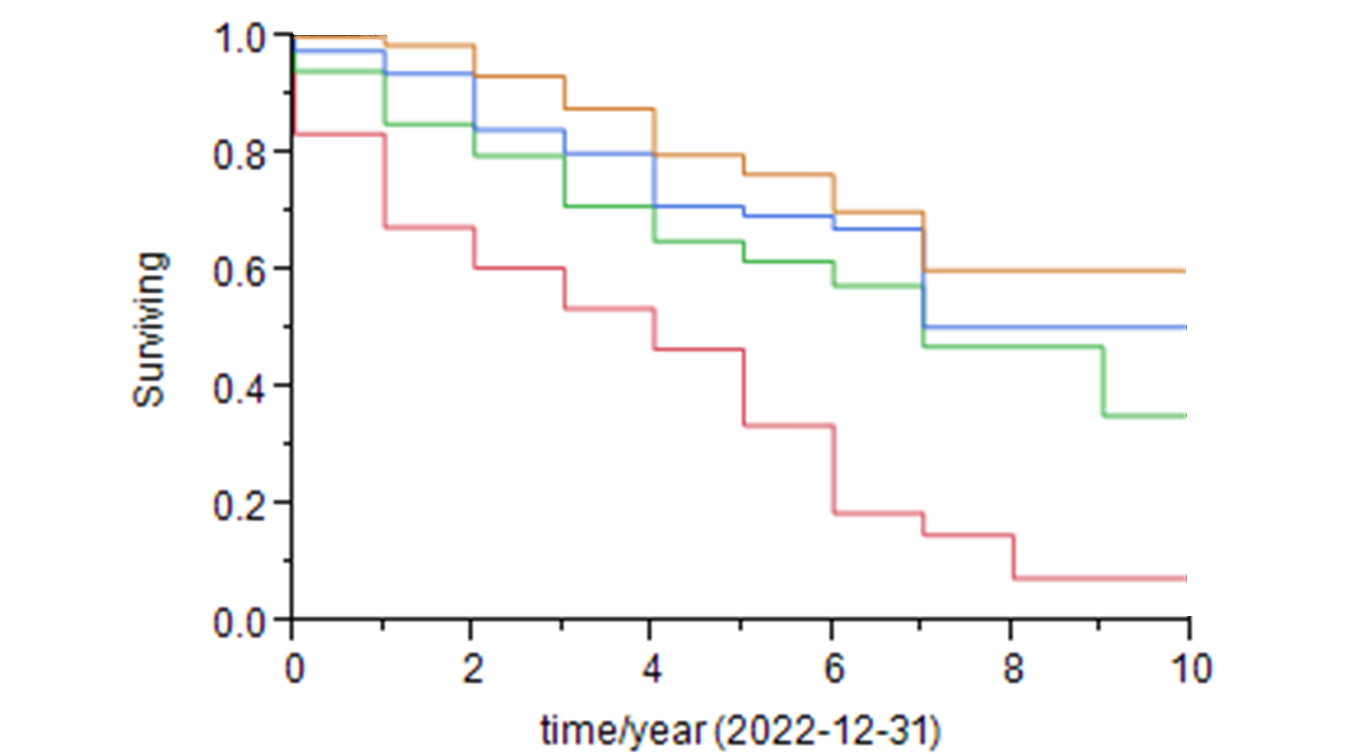


**Q4**

**Q3**

**Q2**

**Q1**

**1.0**

**0.8**

**0.6**

**0.4**

**0.2**

**0.0**

**Overall survival (%)**

**0 2 4 6 8 10**

**Follow-up (years)**

**P <0.0001 by Log Rank**

Supplementary Figure 2. Kaplan-Meier survival curves were plotted to assess all-cause mortality across quartiles of retinol-binding protein 4 levels at baseline. The p-value indicates the significance of the log-rank test conducted across quartiles.

**ROC Curve**


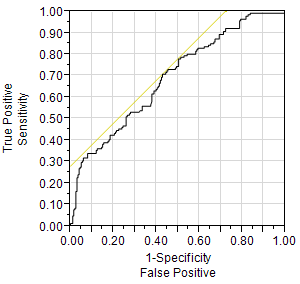


**1.00**

**0.90**

**0.80**

**0.70**

**0.60**

**0.50**

**0.40**

**0.30**

**0.20**

**0.10**

**0.00**

**Sensitivity**

**0.00 0.20 0.40 0.60 0.80 1.00**

**1-Specificity**

**Area Under Curve: 0.692 Criterion <95.62 ug/mL**

**Sensitivity: 70.9%**

**Specificity: 42.8%**

**p-value <0.0001**

Supplementary Figure 3. Receiver operator characteristics (ROC) curve of baseline retinol-binding protein 4 for the detection of all-cause mortality. A threshold value of <95.62 ug/mL is associated with all-cause mortality with a sensitivity of 70.9% and specificity of 42.8%.

Supplementary Table 5. Relationship between baseline plasma retinol-binding protein 4 levels and

clinical and biochemical parameters

| Variable | r | p-value |
| --- | --- | --- |
| Age | -0.295 | <0.0001 |
| Sex | -0.033 | 0.544 |
| Time on hemodialysis | 0.282 | <0.0001 |
| Body mass index | 0.037 | 0.502 |
| Systolic blood pressure | 0.114 | 0.036 |
| Diastolic blood pressure | 0.144 | 0.008 |
| Total cholesterol | 0.178 | 0.001 |
| Triglyceride | 0.209 | 0.0002 |
| Fasting glucose | -0.132 | 0.017 |
| Estimated glomerular filtration rate | -0.397 | <0.0001 |
| Creatinine | 0.152 | 0.005 |
| Uric acid | 0.180 | 0.001 |
| Platelet | 0.123 | 0.026 |
| Hemoglobin | 0.127 | 0.021 |
| Hematocrit | 0.053 | 0.340 |
| Mean corpuscular volume | -0.168 | 0.002 |
| Ferritin | 0.013 | 0.821 |
| Transferrin saturation | 0.030 | 0.599 |
| Sodium | 0.088 | 0.115 |
| Potassium | 0.306 | <0.0001 |
| Calcium | 0.202 | 0.0002 |
| Phosphate | 0.246 | <0.0001 |
| Parathyroid hormone | 0.191 | 0.002 |
| Albumin | 0.431 | <0.0001 |
| Total protein | 0.177 | 0.001 |
| Aspartate aminotransferase | -0.127 | 0.022 |
| Alanine aminotransferase | -0.047 | 0.395 |
| White blood cell | 0.012 | 0.834 |
| Red blood cell | 0.134 | 0.015 |
| High-sensitivity C-reactive protein | -0.197 | 0.001 |
| Unsaturated iron-binding capacity | 0.059 | 0.301 |
| Urea reduction ratio | 0.084 | 0.131 |
| Normalized protein catabolic rate | 0.224 | 0.003 |

Supplementary Table 6. Cox regression analysis of all-cause mortality including interaction terms: baseline RBP4 × hs-CRP and baseline RBP4 × albumin

|  |  | All-cause mortality | | |
| --- | --- | --- | --- | --- |
|  |  | Model 1 | Model 2 | Model 3 |
| Variables |  | HR (95%CI) p-value | HR (95%CI) p-value | HR (95%CI) p-value |
| Baseline RBP4 |  | 0.98 (0.97-0.98) <0.0001 | 0.98 (0.97-0.99) <0.0001 | 0.99 (0.98-1.00) 0.008 |
| Hs-CRP |  | 1.20 (1.06-1.37) 0.005 | 1.15 (1.01-1.32) 0.039 | 1.17 (1.00-1.38) 0.042 |
| RBP4 × hs-CRP |  | 0.99 (0.99-1.00) 0.281 | 0.99 (0.99-1.00) 0.104 | 1.00 (0.99-1.00) 0.313 |
| Baseline RBP4 |  | 0.98 (0.97-0.99) 0.0003 | 0.99 (0.98-1.00) 0.004 | 0.99 (0.98-1.00) 0.023 |
| Albumin |  | 0.27 (0.17-0.43) <0.0001 | 0.33 (0.20-0.55) <0.0001 | 0.38 (0.19-0.76) 0.007 |
| RBP4 × albumin |  | 0.99 (0.98-1.01) 0.685 | 0.99 (0.99-1.02) 0.840 | 0.99 (0.98-1.02) 0.752 |

Model 1: Univariate logistic regression analysis

4.0

Model 2: Adjusted for age and gender

Model 3: Adjusted for age, gender, body mass index, hypertension, diabetes mellitus, triglycerides, estimated glomerular filtration rate, hemoglobin, aspartate aminotransferase, alanine aminotransferase, and uric acid.

HR, hazard ratio; RBP, retinol-binding protein; Hs-CRP, high-sensitivity C-reactive protein.
